# Acyl-CoA thioesterase activity of peroxisomal ABC protein ABCD1 is required for the transport of very long-chain acyl-CoA into peroxisomes

**DOI:** 10.1038/s41598-021-81949-3

**Published:** 2021-01-26

**Authors:** Kosuke Kawaguchi, Emi Mukai, Shiro Watanabe, Atsushi Yamashita, Masashi Morita, Takanori So, Tsuneo Imanaka

**Affiliations:** 1 Graduate School of Medicine and Pharmaceutical Sciences, University of Toyama, 2630 Sugitani, Toyama, 930-0194 Japan; 2 Institute of Natural Medicine, University of Toyama, 2630 Sugitani, Toyama, 930-0194 Japan; 3 Faculty of Pharma-Sciences, Teikyo University, 2-11-1 Kaga, Itabashi-Ku, Tokyo, 173-8605 Japan; 4 Faculty of Pharmaceutical Sciences, Hiroshima International University, 5-1-1 Hirokoshinkai, Kure, Hiroshima 737-0112 Japan

**Keywords:** Biochemistry, Cell biology

## Abstract

The ABCD1 protein, one of the four ATP-binding cassette (ABC) proteins in subfamily D, is located on the peroxisomal membrane and is involved in the transport of very long chain fatty acid (VLCFA)-CoA into peroxisomes. Its mutation causes X-linked adrenoleukodystophy (X-ALD): an inborn error of peroxisomal β-oxidation of VLCFA. Whether ABCD1 transports VLCFA-CoA as a CoA ester or free fatty acid is controversial. Recently, Comatose (CTS), a plant homologue of human ABCD1, has been shown to possess acyl-CoA thioesterase (ACOT) activity, and it is suggested that this activity is required for transport of acyl-CoA into peroxisomes. However, the precise transport mechanism is unknown. Here, we expressed human His-tagged ABCD1 in methylotrophic yeast, and characterized its ACOT activity and transport mechanism. The expressed ABCD1 possessed both ATPase and ACOT activities. The ACOT activity of ABCD1 was inhibited by *p*-chloromercuribenzoic acid (*p*CMB), a cysteine-reactive compound. Furthermore, we performed a transport assay with ABCD1-containing liposomes using 7-nitro-2–1,3-benzoxadiazol-4-yl (NBD)-labeled acyl-CoA as the substrate. The results showed that the fatty acid produced from VLCFA-CoA by ABCD1 is transported into liposomes and that ACOT activity is essential during this transport process. We propose a detailed mechanism of VLCFA-CoA transport by ABCD1.

## Introduction

Peroxisomes are membrane-bound organelles that are found in almost all eukaryotic cells. They are essential to cellular function and involved in a variety of metabolic processes. Their essential activities include the β-oxidation of fatty acids, especially very long chain fatty acids (VLCFA, > C22), as well as the synthesis of bile acid and plasmalogen^1–3^. In these metabolic pathways, metabolites must be transported efficiently in and out of peroxisomes. Recent studies have revealed that such metabolite transport is facilitated by at least several different membrane-bound transporters^4,5^. One of the transporter families is the ATP-binding cassette (ABC) proteins. They transport a wide variety of substrates, including lipids, across membranes in an ATP-dependent manner. In humans, 48 ABC proteins have been identified and divided into seven subfamilies, A to G, based on similarities in gene organization and sequence.

Peroxisomal ABC proteins in humans are classified into “subfamily D” and three ABCD proteins, ABCD1‒3, have been identified^6–10^. Dysfunction of ABCD1 causes neurodegenerative disorder X-linked adrenoleukodystrophy (X-ALD), which is characterized by the abnormal accumulation of VLCFA due to impaired peroxisomal β-oxidation. Therefore, ABCD1 is required for the VLCFA β-oxidation pathway in peroxisomes^11^. ABCD1 and ABCD2 are suggested to be involved in the transport of long chain fatty acid (LCFA)-CoA and VLCFA-CoA into peroxisomes with different substrate specificities, while ABCD3 is involved in the transport of branched chain acyl-CoA and the bile acid intermediates di- and tri-hydroxycholestanoyl-CoA^12–14^. However, the precise transport mechanism has yet to be elucidated.

In early studies, the transfection of human *ABCD1* cDNA into X-ALD skin fibroblasts restored VLCFA β-oxidation activity and the VLCFA contents was back to normal in the fibroblasts. Therefore, ABCD1 was thought to function as a transporter of VLCFA across the peroxisomal membranes^15,16^. Subsequently, van Roermund et al. further demonstrated that human ABCD1 is involved in the transport of VLCFA-CoA across the peroxisomal membranes by expressing human ABCD1 in *Saccharomyces cerevisiae*^12,17^. In fact, Ofman et al. have showed that the C24:0-CoA and C26:0-CoA levels in X-ALD fibroblasts were increased when incubated with C24:0^18^. These findings strongly suggest that ABCD1 plays an important role in the uptake of a wide range of VLCFA-CoA isoforms into peroxisomes. However, how acyl-CoA is transported into peroxisomes has not been characterized to date.

In the case of yeast cells, disruption of *PXA1* and/or *PXA2*, a heterodimer of the homolog of human ABCD1, resulted in impaired growth of these mutants on oleic acid as the sole carbon source, and also a reduced ability to oxidize oleate^19^. In addition, Verleur et al. used a semi-intact yeast cell system to show that Pxa1p and Pxa2p are directly responsible for the ATP-dependent transport of LCFA-CoA across peroxisomal membranes^20^. These results clearly indicate that Pxa1p/Pxa2p functions as a transporter of acyl-CoA. In addition, one of the approaches to determining whether acyl-CoA undergoes hydrolysis before or during the translocation of peroxisomes is to measure the incorporation of ^18^OH from [^18^O] H_2_O in the course of the incubation of yeast cells with acyl-CoA. van Roermund et al. demonstrated that substrate acyl-CoA was hydrolyzed and the carboxyl group of acyl-CoA was labeled with ^18^O before re-esterification to the corresponding CoA ester^21^. However, the protein responsible for the acyl-CoA thioesterase (ACOT) activity has not been identified.

In the case of plants, the double mutation of *lacs6-1* and *lacs7-1*, which encode peroxisomal LCFA-CoA synthetase isozymes in *Arabidopsis thaliana*, was shown to result in a similar phenotype to mutations in Comatose (CTS), a homologue of human ABCD1, in terms of LCFA β-oxidation. It was postulated that peroxisomal LCFA-CoA synthetase is needed if CTS delivers unesterified fatty acids into the peroxisomal matrix^22^. Thereafter, De Marcos Lousa et al. demonstrated that CTS has an intrinsic ACOT activity and suggested that LCFA-CoA is hydrolyzed prior to transport in plant cells^23^. On the other hand, Wiesinger et al. found that β-oxidation of VLCFA-CoA in isolated peroxisomes from human fibroblasts did not change with or without addition of CoA and suggested that VLCFA-CoA was transported directly^24^. Therefore, whether ABCD proteins translocate acyl-CoAs or free fatty acids derived from its CoA ester by hydrolysis is still controversial and a precise characterization of the transport mechanism is required.

We previously established a procedure for expressing human ABCD1 in the methylotrophic yeast *Komagataella phaffii* (formerly called *Pichia pastoris*), purifying ABCD1 and reconstituting the detergent-solubilized protein into liposomes, and demonstrated that human ABCD1 has ACOT activity^25^. In this study, we analyzed the properties of the ACOT activity of human ABCD1. Furthermore, we have developed a novel and concise transport assay using 7-nitro-2–1,3-benzoxadiazol-4-yl (NBD)-labelled acyl-CoA as a substrate and directly demonstrated that ABCD1 employed fatty acyl-CoA as a substrate for transport, and the fatty acid moiety of the ABCD1-catalyzed ACOT activity was transported into ABDC1-liposomes. These results indicate that ABCD1-catalyzed ACOT activity is coupled to and indispensable for the transport by ABCD1.

## Results

### Human ABCD1 possesses both ATPase and ACOT activities

**Figure 1:**
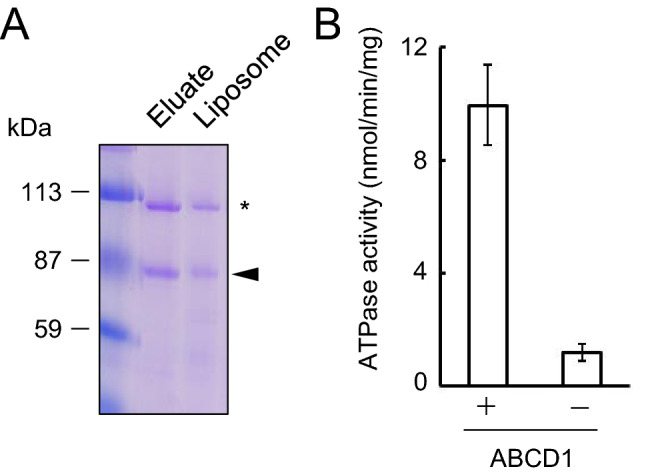
ATPase activities of reconstituted ABCD1. (**A**) Purified His-ABCD1 (Eluate) and reconstituted His-ABCD1 (Liposome) were subjected to SDS-PAGE, and the gels were stained with CBB. The arrow head and the asterisk indicate His-ABCD1 and a non-specific protein, respectively. (**B**) ATPase activity of reconstituted ABCD1 was measured. Proteoliposomes containing ABCD1 (3.05 μg) or negative control liposomes containing non-specific protein (1.98 μg) were incubated with 5 mM ATP for 30 min at 37 °C and the phosphate that was released was measured. Error bars indicate the standard error (n = 3).

We previously demonstrated that human ABCD1 possesses ACOT activity similar to CTS, a homologue of human ABCD1 in *A. thaliana*^25^. To further characterize ACOT activity of human ABCD1, we first prepared the *Kppxa1*Δ strain that has lost the endogenous peroxisomal ABC transporter (Fig. S1). Then His-tagged human ABCD1 (His-ABCD1) was expressed under the control of the potent methanol-inducible *AOX1* promoter in the *Kppxa1*Δ strain. Utilizing the procedure we previously established^25^, the His-ABCD1 expressed in *K. phaffii* was solubilized by *n*-dodecyl-β-d-maltoside (β-DDM) and then purified using a metal-chelate resin. Subsequently, purified His-ABCD1 was reconstituted into liposomes (Fig. 1A). As ABCD1 exists on liposomes in both an inside-out and right-side-out orientation, we evaluated the proportion of the right-side-out orientation. ABCD1-liposomes were treated with trypsin and subjected to immunoblot analysis using anti-ABCD1 antibody recognizing C-terminal half of ABCD1. As shown in Fig. S2A, approximately 33 kDa band emerged. This band seems to be derived from ABCD1 existing in inside-out orientation. The ratio of inside-out to right-side-out was approximately 1:1 calculated from the signal intensities (Fig. S2B). The 110 kDa band in the reconstituted liposomes is a non-specific protein derived from the yeast and is reconstituted into liposomes^25^, so the proteoliposomes containing the non-specific protein were used as a negative control (Fig. S3). Employing the ABCD1-reconstituted liposomes, the ATPase and ACOT activities in ABCD1 were further characterized. ABCD1-liposomes possess ATPase activity, but such activity was absent in the negative control liposomes, suggesting that the active ABCD1 had been purified and reconstituted. (Fig. 1B).

**Figure 2:**
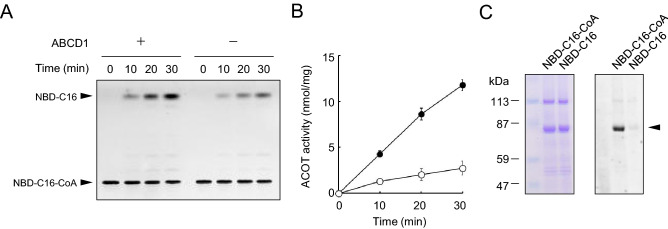
ACOT activities of reconstituted ABCD1. (**A**) Thioesterase activity of reconstituted ABCD1. Proteoliposomes containing ABCD1 (1.84 μg) or negative control liposomes containing non-specific protein (1.62 μg) were incubated with 2 μM NBD-C16-CoA for 10, 20, and 30 min at 37 °C. The “no incubation (0 min)” was run as the reaction was stopped before the addition of NBD-C16-CoA. Each reaction was subjected to TLC to separate NBD-C16-CoA and NBD-C16. (**B**) The ACOT activity was determined after the amount of NBD-C16 was quantified using the image analysis software Image J, and the ACOT activity of ABCD1 (filled circle) and non-specific protein (open circle) are shown. Error bars indicate the standard error (n = 3). (**C**) Acylation of ABCD1 with NBD-C16-CoA. ABCD1-liposomes were incubated with NBD-C16-CoA or NBD-C16 for 30 min at 37 °C and then subjected to SDS-PAGE. The gel was fixed with methanol and stained with CBB (left panel) after the detection of NBD fluorescence (right panel). The arrow head indicates ABCD1.

We developed the assay for the ACOT activity using fluorescent substrate NBD-C16-CoA. Because ABCD1 is known to be involved in the processing of VLCFA, NBD-C16-CoA is employed as a VLCFA-CoA analogue. The addition of the NBD moiety to the omega carbon of palmitate results in a similar size and hydrophobicity as that of VLCFA. ABCD1-liposomes were incubated with 2 μM NBD-C16-CoA, then the substrate and product were separated by thin layer chromatography (TLC) (Fig. 2A,B). NBD-C16 formed in a time-dependent manner. Because the formation of NBD-C16 in the negative control liposomes was low, the formation of NBD-C16, i.e., the hydrolysis of NBD-C16-CoA, is due to ABCD1. These results indicate that ABCD1 catalyzes ACOT activity.

We next examined the mechanism of the reaction. We investigated whether ABCD1 forms an intermediate with NBD-C16-CoA. ABCD1-liposomes were incubated with NBD-C16-CoA, then subjected to SDS-PAGE. As shown in Fig. 2C, the 80 kDa band corresponding to ABCD1 exhibited NBD fluorescence, demonstrating that ABCD1 is covalently labeled by NBD-C16. ABCD1 was not labeled when incubated with NBD-C16, suggesting that the activation of NBD-C16 (NBD-C16-CoA) is needed for the acylation (the formation of the ABCD1-NBD-C16 intermediate). These results suggest that the ABCD1-catalyzed ACOT activity occurs as the result of following three steps; (i) the binding of acyl-CoA to the ACOT domain of ABCD1, (ii) acylation of the amino acids located in the active cite of ACOT with fatty acids (i.e., the formation of the fatty acid-ACOT intermediate) and iii) hydrolysis of the fatty acid ester of the intermediate.

### The amino acid residues responsible for the ACOT activity of ABCD1

**Figure 3:**
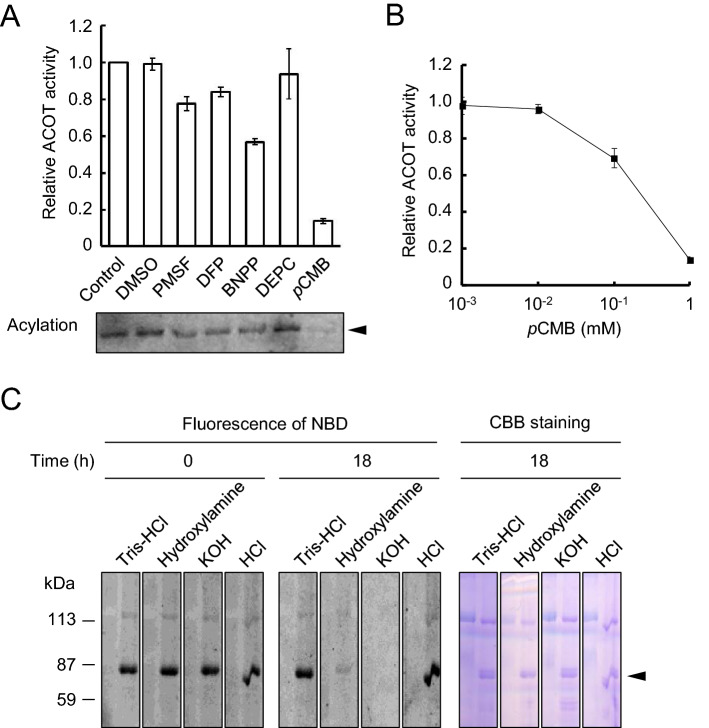
Amino acid residues responsible for the ACOT activity of ABCD1 (**A**) Proteoliposomes containing ABCD1 (2.70 μg) were incubated with NBD-C16-CoA in the presence or absence of each compound (1 mM) for 30 min at 37 °C. Aliquots were subjected to TLC as shown in Fig. S4A. Relative ACOT activities were evaluated by quantifying the intensities of NBD-C16 using the image analysis software Image J (upper graph). ACOT activity without any reagents (0.27 nmol/min/mg) has been normalized to 1. The aliquots were also subjected to SDS-PAGE and the acylation of ABCD1 with NBD-C16-CoA was analyzed (lower panel). Error bars indicate the standard error (n = 3). The arrow head indicates ABCD1. PMSF: phenylmethylsulfonyl fluoride, DFP: diisopropylfluorophosphate, BNPP: bis-(4-nitrophenyl)phosphate, *p*CMB: *p*-chloromercuribenzoic acid, DEPC: diethyl pyrocarbonate. (**B**) ACOT activity was measured in the presence of various concentrations of *p*CMB for 30 min at 37 °C. Aliquots were subjected to TLC as shown in Fig. S4B. Relative ACOT activities were evaluated by quantifying the intensities of NBD-C16 using the image analysis software Image J. ACOT activity without *p*CMB (0.29 nmol/min/mg) has been normalized to 1. (**C**) Stability of covalent linkage between ABCD1 and NBD-C16. ABCD1-liposomes were incubated with NBD-C16-CoA for 30 min at 37 °C and then subjected to SDS-PAGE. The gels were fixed and then divided. Each gel was incubated with 1 M Tris–HCl pH 7.0, 1 M Hydroxylamine pH 7.0, 0.1 N KOH or 0.1 N HCl for 18 h at room temperature, respectively. The gels were stained with CBB after the detection of NBD fluorescence. The arrow head indicates ABCD1.

To determine the amino acid residues involved in the active site of ACOT activity in ABCD1, we evaluated the effects of several chemical modifiers on the ACOT activity of ABCD1. ABCD1-liposomes were incubated with NBD-C16-CoA together with 1 mM of common amino acid modifying reagents. The cysteine-reactive reagent *p*-chloromercuribenzoic acid (*p*CMB) strongly decreased the ABCD1-catalyzed ACOT activity to 13.8 ± 1.41%. Simultaneously, the acylation of ABCD1 disappeared (Figs. 3A, S4A). This inhibition was observed in a dose-dependent manner over 10 μM of *p*CMB (Figs. 3B, S4B). The results indicate that modification of the cysteine residue(s) inhibits the acylation of the intrinsic activity of ABCD1 (the formation of ABCD1-NBD-C16 intermediate (the above-mentioned step ii)). The stability of the covalent linkage between ABCD1 and NBD-C16 was tested to further evaluate the involvement of cysteine residue in the formation of the intermediate during ABCD1-catalyzed ACOT activity. After incubation with NBD-C16-CoA, ABCD1-liposomes were subjected to SDS-PAGE. Subsequently, the acrylamide gel was incubated with 0.1 N HCl, 0.1 N KOH, 1 M hydroxylamine pH 7.0 or 1 M Tris buffer pH 7.0 for 18 h. Treatment with either HCl or Tris–HCl did not decrease the NBD-labeling of ABCD1, whereas KOH or hydroxylamine significantly removed the covalently bound NBD-C16 (Fig. 3C). The disappearance of NBD-labeling in the presence of KOH indicates that NBD-C16 is esterified at a particular amino acid. Furthermore, the label was eliminated by hydroxylamine treatment, indicating the covalent binding is a thioester linkage. Collectively, the covalent intermediate is due to the thioester linkage between the fatty acid and cysteine residue(s) of ABCD1.

In addition, the serine esterase inhibitors phenylmethylsulfonyl fluoride (PMSF), diisopropylfluorophosphate (DFP) and bis-(4-nitrophenyl)phosphate (BNPP) reduced the ABCD1-catalyzed ACOT activity to 77.4 ± 3.76%, 83.9 ± 2.75% and 56.7 ± 1.54%, respectively. However, acylation of ABCD1 was still observed (Fig. 3A, lower panel). These results may indicate that the modification of serine residue(s) mainly inhibit the intrinsic activity of the hydrolysis of ABCD1-NBD-C16 intermediate (the above-mentioned step iii)). The histidine-reacting reagent diethyl pyrocarbonate (DEPC) had no effect on the ABCD1-catalyzed ACOT activity (Figs. 3A, S4B), suggesting that the histidine residue is not involved in ACOT activity. Based on these results, it is suggested that the cysteine residue(s) plays an important role in the ABCD1-catalyzed ACOT activity.

### The substrate recognition mechanism of ABCD1-catalyzed ACOT activity

**Figure 4:**
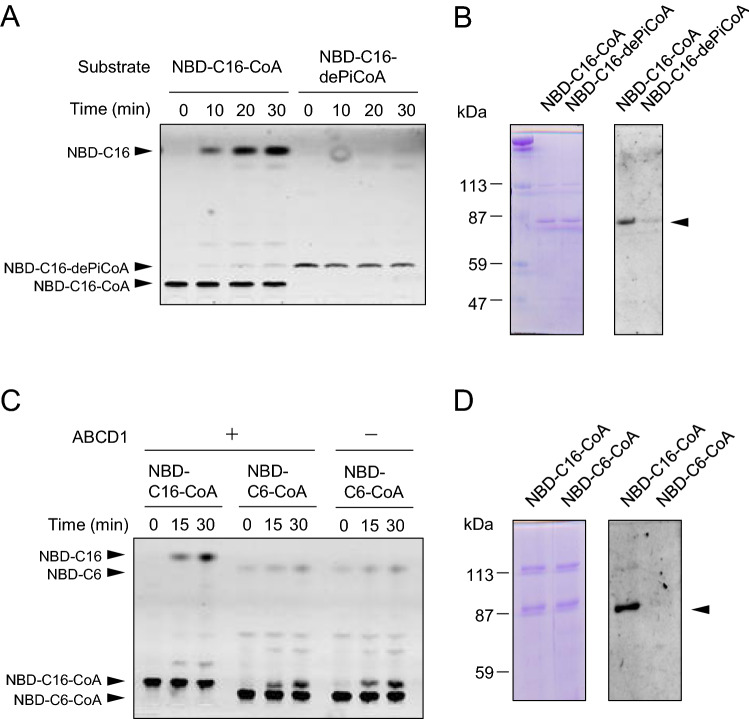
Substrate specificity of ABCD1-catalyzed ACOT activity. (**A**) Proteoliposomes containing ABCD1 (3.04 μg) were incubated with NBD-C16-CoA or NBD-C16-3′-dephosphoCoA (NBD-C16-dePiCoA) for 30 min at 37 °C. The aliquots were subjected to TLC. (**B**) Aliquots were also subjected to SDS-PAGE. The gel was fixed with methanol and stained with CBB (left panel) after the detection of NBD fluorescence (right panel). The arrow head indicates ABCD1. (**C**) Proteoliposomes containing ABCD1 (3.16 μg) were incubated with NBD-C16-CoA or NBD-C6-CoA for 30 min at 37 °C. The aliquots were subjected to TLC. (**D**) Aliquots were also subjected to SDS-PAGE. The gel was fixed with methanol and stained with CBB (left panel) after the detection of NBD fluorescence (right panel). The arrow head indicates ABCD1.

As mentioned above, ABCD1 is acylated by NBD-C16 in the course of the hydrolysis of NBD-C16-CoA. Covalent modification by fatty acylation occurs in a variety of viral and cellular proteins (e.g. hemagglutinin^26^, phospholipase A_2_γ^27^, neuromodulin^28^). In the case of UDP-glucuronosyltransferase (UGT), UGT is acylated by palmitoyl-CoA, but not by palmitoyl-3′-dephosphoCoA, an analogue lacking the 3′- phosphate group on the ribose moiety, suggesting the 3′-phosphate is important for substrate recognition^29^. Therefore, we tested whether the ABCD1-catalyzed ACOT activity recognizes NBD-C16-3′-dephosphoCoA as a substrate. NBD-C16-3′- dephosphoCoA was prepared by Nuclease P1 treatment of NBD-C16-CoA. As shown in Fig. 4A, ABCD1 was unable to release NBD-C16 from NBD-C16-3′-dephosphoCoA. Furthermore, NBD-C16-3′-dephosphoCoA also did not serve as a substrate for the acylation of ABCD1 (Fig. 4B). Meanwhile, NBD-C16-3′-dephosphoCoA was hydrolyzed by esterase from porcine liver (Fig. S5). These results indicate that ABCD1 strictly recognizes the 3′-phosphate on ribose ring of fatty acyl-CoA as substrate for the formation of acyl-ABCD1 intermediate before subsequent hydrolysis, although the esterase in porcine liver does not possess specific substrate recognition.

Since the carbon chain length of fatty acyl-CoA is also considered to be important for the substrate specificity of ABCD1, we tested whether a shorter chain length of fatty acyl-CoA would serve as a substrate. We employed NBD-hexanoic acid (NBD-C6) as an analogue for the medium to long chain fatty acid. To prepare NBD labeled fatty acyl-CoA, we first constructed His-tagged *K. phaffii* FAA2 (His-*Kp*FAA2), an acyl-CoA synthetase expressing *K. phaffii* SMD1168 strain. Subsequently, His-*Kp*FAA2 was purified using the same procedure as used for His-ABCD1 (Fig. S6). NBD-hexanoic acid (NBD-C6) was incubated with purified His-*Kp*FAA2 to generate NBD-hexanoyl-CoA (NBD-C6-CoA).

When ABCD1-liposomes were incubated with NBD-C6-CoA, the release of NBD-C6 was not observed, suggesting that NBD-C6-CoA did not serve as a substrate for the ABCD1-catalyzed ACOT activity (Fig. 4C). Furthermore, acylation of ABCD1 was not observed with NBD-C6-CoA (Fig. 4D). These results showed that ABCD1 does not recognize NBD-C6-CoA as a substrate. Collectively, these findings clearly indicate that both 3′-phosphate on the ribose ring of the CoA moiety and the fatty acid chain length determine the substrate specificity of acyl-CoA for ABCD1-catalyzed ACOT activity.

### The relationship between ATPase and the ACOT activities of human ABCD1

**Figure 5:**
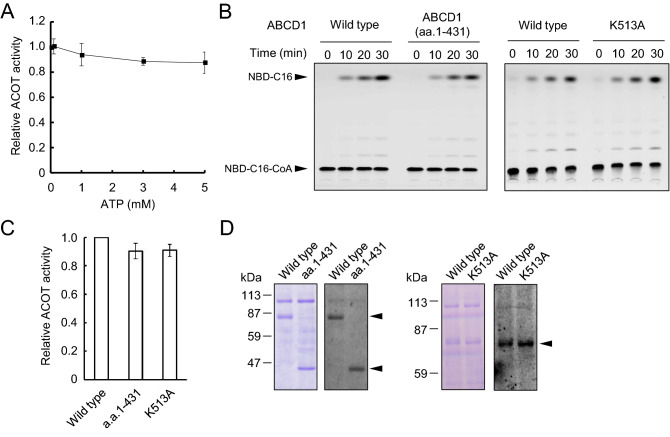
Relationship between ATPase activity and the ACOT activity of ABCD1 (**A**) Effect of ATP on ABCD1-catalyzed ACOT activity. ABCD1-liposomes were incubated with NBD-C16-CoA in the presence of various concentration of ATP for 30 min at 37 °C. The aliquots were subjected to TLC as shown in Fig. S7. The relative ACOT activities were evaluated by quantifying the intensities of NBD-C16 using the image analysis software Image J. Error bars indicate the standard error (n = 3). (**B**) His-ABCD1(a.a.1–431) and His-ABCD1(K513A) were prepared using the same procedure as the purification of His-ABCD1 and then reconstituted into liposomes. Proteoliposomes containing ABCD1wild type, ABCD1(K513A) or ABCD1(1–431) (2.68 μg, 3.21 μg or 3.71 μg, respectively) were incubated with NBD-C16-CoA for 30 min at 37 °C. The aliquots were subjected to TLC to separate hydrolyzed NBD-C16 and NBD-C16-CoA. (**C**) Relative ACOT activities of ABCD1(a.a.1–431) and ABCD1(K513A) were evaluated. The intensities of NBD-C16 were normalized by the signal intensities of each of the proteins obtained by immunoblot analysis using the image analysis software Image J. Error bars indicate the standard error (n = 3). Wild type activity (0.33 nmol/min/mg) has been normalized to 1. (**D**) Aliquots were also subjected to SDS-PAGE. The gel was fixed with methanol and stained with CBB (left panel) after the detection of NBD fluorescence (right panel). The arrow heads indicate the wild type, a.a.1–431 or K513A.

In the case of CTS, ACOT activity is stimulated in the presence of ATP and is dependent on ATPase activity^23,30^. We first evaluated the effect of ATP on the ACOT activity of human ABCD1. ABCD1-liposomes were incubated with NBD-C16-CoA in the presence of the indicated concentration of ATP. However, the ACOT activity of ABCD1 was not stimulated by ATP (Figs. 5A, S7).

Furthermore, to test whether ACOT activity is dependent on ATPase activity or not, we prepared mutant ABCD1s. These were ABCD1(a.a.1‒431), containing only the N-terminal and the transmembrane domain composed of six transmembrane helices without the nucleotide binding domain, as deduced by a structural model of ABCD1 based on ABCB10 homology^31^, and ABCD1(K513A), a Walker A lysine mutant deficient in ATPase activity as well as other ABC transporters^32–34^. We also expressed His-ABCD1(a.a.1‒431) and His-ABCD1(K513A) under the control of the *AOX1* promoter in the *Kppxa1*Δ strain. His-ABCD1(a.a.1‒431) and His-ABCD1(K513A) were purified and reconstituted into liposomes using the same procedure as used for His-ABCD1 (Fig. S8A).

Since ABCD1(a.a.1‒431) does not contain the nucleotide binding domain and ABCD1(K513A) is mutated in the Walker A motif, both ABCD1(a.a.1‒431) and ABCD1(K513A) are unable to hydrolyze ATP. However, these mutants possess ACOT activity comparable to the wild type and exhibited covalent linkage with the substrate (Fig. 5B‒D). These results indicate that ATPase activity is not essential for ABCD1-catalyzed ACOT activity. Furthermore, it is suggested that the active site of ACOT activity exists in the N-terminal and transmembrane domain composed of six transmembrane helices^31^, since ABCD1(a.a.1‒431) possesses ACOT activity corresponding with wild type ABCD1 (Fig. 5B,C). These results strongly indicate that the ACOT activity of ABCD1 is independent of the ATPase activity. In the case of CTS in *A. thaliana*, the ACOT activity is dependent on the ATPase activity, but not vice versa^30^.

### Transport of NBD-palmitic acid into ABCD1-liposomes

A precise evaluation of the transport of hydrophobic molecules into vesicles is generally difficult because of the nature of the hydrophobic molecules, including LCFA, VLCFA and their CoA esters, as there is a tendency to be adsorbed on the surface of the vesicle membrane. It is difficult to distinguish between the substrate(s) which are transported and localize inside of the vesicle and those absorbed on the outside of the membrane surface. We established a transport assay into ABCD1-liposomes using NBD-C16-CoA as the substrate. The NBD-C16-CoA remaining outside or on the outer-surface of ABCD1-liposomes was quenched with sodium dithionite to reduce the excess amount of substrate remaining after the transport as described previously^35^.

**Figure 6:**
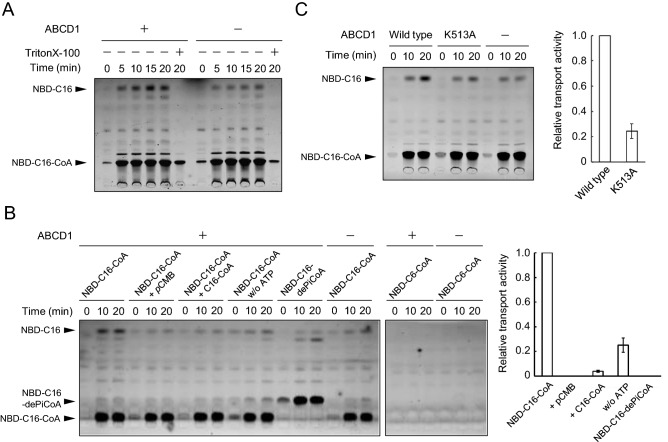
Transport of the NBD-C16 derived from NBD-C16-CoA into ABCD1- liposomes. (**A**) Proteoliposomes containing ABCD1 (4.81 μg) or non-specific protein were incubated with NBD-C16-CoA at 37 °C for the indicated periods. After incubation, the remaining NBD-C16-CoA in the outside portion of the liposomes was quenched with sodium dithionite. In some cases, the liposomes were treated with sodium dithionite after the addition of 0.01% Triton X-100. Subsequently, ABCD1-liposomes were precipitated by centrifugation and then resuspended with 80% acetone. NBD-C16 and NBD-C16-CoA were separated by TLC. (**B**) Transport of NBD-C16 into ABCD1-liposomes was tested under various conditions. ABCD1-liposomes were incubated with NBD-C16-CoA in the presence of *p*CMB (1 mM) or palmitoyl-CoA (20 μM), or in the absence of ATP. NBD-C16-3′-dephosphoCoA (NBD-C16-dePiCoA) and NBD-C6-CoA were also examined as the substrate. Relative NBD-C16 transport activities were evaluated by quantifying the intensities of NBD-C16 using the image analysis software Image J (right graph). Error bars indicate the standard error (n = 3). (C) NBD-C16 transport activity for ABCD1(K513A) was tested by the same procedure as used for the wild type. The relative transport activity ABCD1(K513A) was evaluated as follows. The intensities of NBD-C16 were normalized by the signal intensities of the wild type and K513A obtained by immunoblot analysis using the image analysis software Image J. Error bars indicate the standard error (n = 3).

ABCD1-liposomes were incubated with NBD-C16-CoA and ATP at 37 °C, and then incubated with sodium dithionite for 15 min on ice. NBD-labeled compounds were extracted with 80% acetone and separated by TLC. Both NBD-C16 and NBD-C16-CoA were detected (Fig. 6A). The signal intensity of NBD-C16 increased in a time-dependent manner. NBD-C16 was quenched when the liposomes were disrupted with Triton X-100 before sodium dithionite treatment (Fig. 6A). These results indicate that NBD-C16 was locate inside or on the inner leaflet of the liposomes. However, NBD-C16 was significantly reduced when employing liposomes containing the non-specific protein (Fig. 6A), and was not observed when employing liposomes without any proteins (Fig. S9B). To demonstrate NBD-C16 was bound to ABCD1 and then released into liposomes during this transport system, the turnover of acylated ABCD1 was tested. ABCD1-liposomes were incubated with NBD-C16-CoA and then free NBD-C16-CoA was removed by size exclusion chromatography. The resulted ABCD1-liposomes were incubated with excess amount of palmitoyl-CoA and the acylation of ABCD1 was examined. As shown in Fig. S9C, the amount of acylated ABCD1 with NBD-C16 was reduced in the time-dependent manner. These results indicate that the NBD-C16 was bound with ABCD1 and released into inside or on the inner leaflet of the ABCD1-liposomes due to the activity of ABCD1.

A significant amount of NBD-C16-CoA was detected on ABCD1-liposomes even after the quenching treatment with sodium dithionite. However, the amount was the same at each time point, and did not depend on the presence of ABCD1 (Fig. 6A). Furthermore, NBD-C16-CoA was also detected even when incubated with liposomes that did not contain any proteins (Fig. S9B). The presence of NBD-C16-CoA in the liposomes is considered to be due to a non-specific event. The balance in the polar (CoA) and nonpolar (NBD-C16) forms may determine the embedding of the NBD moiety in the outer leaflet of the liposomes. The embedding of the NBD moiety results in a reduced quenching of the NBD moiety of NBD-C16-CoA because of the reduced access of hydrophilic dithionite to the NBD moiety in the hydrophobic environment.

The transport properties of ABCD1 were examined with a transport assay. The presence of *p*CMB completely abolished the transport of NBD-C16 into ABCD1-liposomes (Fig. 6B). Because *p*CMB inhibits ABCD1-catalyzed ACOT activity (Fig. 3A,B), the inhibition of hydrolysis resulted in the inhibition of the transport. An excess amount of palmitoyl-CoA significantly reduced the amount of NBD-C16 transported into ABCD1-liposomes to 3.95 ± 0.70% (Fig. 6B). This result is due to the competitive inhibition of the hydrolysis of NBD-C16-CoA and transport of NBD-C16 by excess palmitoyl-CoA. When ABCD1-liposomes were incubated with NBD-C16-3′-dephosphoCoA or NBD-C6-CoA, which are not hydrolyzed by ABCD1, the NBD-labeled fatty acids were not transported into ABCD1-liposomes (Fig. 6B). These results indicate that NBD-C16-CoA was hydrolyzed by ABCD1 and then the resultant fatty acid moiety was transported into ABCD1-liposomes. NBD-C16-3′-dephosphoCoA was also detected with a similar level of NBD-C16-CoA (Fig. 6B), suggesting that this result was also due to the non-specific event mentioned above. However, NBD-C6-CoA was not detected in a similar procedure (Fig. 6B). The reason why sodium dithionite was able to completely quench the NBD-C6-CoA is considered to be that NBD-moiety exists outside of the liposomes, or is shallowly embedded in the outer leaflet, because the ratio of polar and nonpolar NBD-C6-CoA is smaller than that of NBD-C16-CoA. To investigate this, the NBD-C16-CoA embedded in the outer leaflet of the liposomes was treated with alkaline (NaOH). The treatment caused complete hydrolysis of NBD-C16-CoA (Fig. S9D), suggesting that the thioester linkage of NBD-C16-CoA is exposed to the outer surface of the liposomes and the NBD-C16 moiety embeds in the outer leaflet of the liposomes.

Although the ABC transporter is known to be dependent on ATP, approximately 30% of the transport activity in ABCD1-liposomes was observed in the absence of ATP (Fig. 6B). In addition, similar transport activity was also observed in ABCD1(K513A)-liposomes (approximately 30% compared with wild type, Fig. 6C). The transport activity was present even in the absence of the consumption of ATP, but the conformational change of the nucleotide binding domain by ATP enhanced the transport activity of ABCD1.

## Discussion

Human peroxisomes have an important role in the β-oxidation of VLCFA, and the mutation of ABCD1 causes a neurodegenerative disease, X-ALD. ABCD1 is suggested to be involved in the transport of VLCFA-CoA, but the precise mechanism of the transport has yet to be elucidated. In the present study, we expressed human ABCD1 in *K. phaffii*, purified and reconstituted ABCD1 in liposomes, and examined the transport mechanism of VLCFA into the liposomes using NBD-C16-CoA. We employed NBD-C16-CoA as a VLCFA-CoA analogue, because the addition of the NBD moiety to the omega carbon of palmitate resulted in a similar size and hydrophobicity of VLCFA.

**Figure 7:**
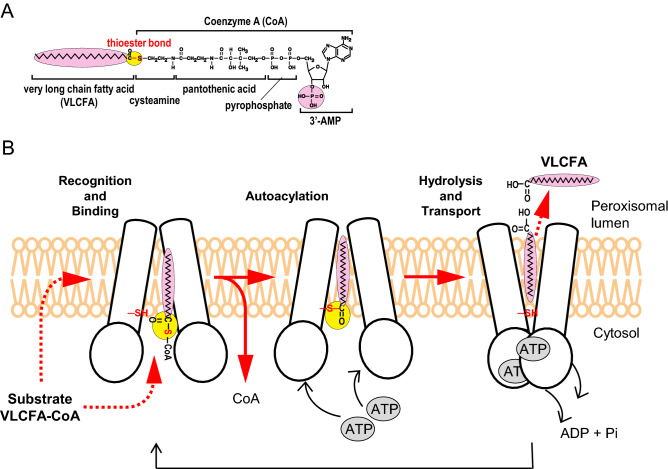
A hypothetical model of VLCFA-CoA transport by ABCD1. (**A**) Structure of VLCFA-CoA. ABCD1 strictly recognizes the VLCFA moiety and 3′-phosphate in the ribose ring of acyl-CoA, which are shown with a pink background. The thioester bond is shown with a yellow background. (**B**) Possible mechanism and the essential roles of acyl-CoA thioesterase activity in the transport of VLCFA by ABCD1. The transport is considered to have three steps; (i) the recognition and binding of VLCFA-CoA to the ACOT domain of ABCD1, (ii) acylation (covalent binding) of the VLCFA moiety to ABCD1, and (iii) the hydrolysis and release of VLCFA from ABCD1 with a conformational change of ABCD1 induced by ATP binding and hydrolysis. The peroxisome membrane is shown in orange. The upper or lower areas of the membrane are inside or outside of the peroxisomes, respectively.

We obtained following the evidence. (1) ABCD1 uses NBD-C16-CoA as a substrate for the transport into liposomes. (2) Free NBD-C16 fatty acid but not NBD-C16-CoA is transported into the liposomes through the hydrolysis of NBD-C16-CoA by the ACOT activity of ABCD1 itself (Fig. 6A). (3) ABCD1 strictly recognizes the 3′-phosphate group on the ribose ring of CoA and the size or hydrophobicity of the fatty acid as substrate, since NBD-C16-3′-dephosphoCoA and NBD-C6-CoA did not serve as a substrate for transport (Fig. 6B). (4) The transport activity was competitively inhibited by an excess amount of palmitoyl-CoA (Fig. 6B). (5) The transport activity of ABCD1 was dependent on ATP and ATPase activity, although the ACOT activity was independent of the ATPase activity (Figs. 5 and 6). (6) The transport of NBD-C16 completely disappeared in the presence of *p*CMB, an inhibitor of the ACOT activity of ABCD1 (Figs. 3B, 6B). Considering these findings, ABCD1 transports VLCFA-CoA as free VLCFA into peroxisomes through the hydrolysis of VLCFA-CoA mediated by the ACOT activity of ABCD1. The transport is considered to be composed of three steps; (i) the recognition and binding of VLCFA-CoA to the ACOT domain of ABCD1, (ii) acylation (covalent binding) of the VLCFA moiety to ABCD1, and (iii) the hydrolysis and release of VLCFA from ABCD1 (Figs. 7B, S9C). The last two steps result in the ACOT activity of ABCD1**.** A recent study of Pxa1p and Pxa2p in *S. cerevisiae* and CTS in *A. thaliana* suggested that these ABC proteins, homologues of human ABCD1, transport free fatty acids after the hydrolysis of acyl-CoA^21,23,30^. Therefore, the transport mechanism is partially conserved in the ABCD protein across species.

Concerning the requirement of the ATPase activity of ABCD1 for the transport of the substrate, we measured the transport of NBD-C16 into ABCD1-liposomes using NBD-C16-CoA as the substrate and also analyzed an ATPase-deficient ABCD1(K513A). In the absence of ATP, the transport activity was dramatically reduced, but ~ 30% of the transport activity remained in the absence of ATP. Similar results were obtained with the ATPase-deficient ABCD1(K513A) (Fig. 6B,C). A portion of NBD-C16 might be translocated from the outer leaflet to the inner leaflet of the liposomes, or released inside of the liposomes without any conformational change of the nucleotide binding domain of ABCD1.

We also found the following characteristics of the ACOT activity of ABCD1. (1) ACOT activity is presumed to locate in the N-terminal and transmembrane domain composed of the six transmembrane helices of ABCD1, and certain cysteine residue(s) are important for the activity (Figs. 3, 5B). (2) The ACOT activity is not stimulated by ATP and is independent of ATPase activity (Fig. 5). (3) The 3′-phosphate group on the ribose ring of CoA and appropriate hydrophobicity of the fatty acid chain are important for the substrate recognition of the ACOT of ABCD1 (Fig. 4A). (4) The ACOT activity of ABCD1 is essential for the transport of NBD-C16-CoA (Fig. 6B).

With regard to the ACOT activity of ABCD1, the sulfhydryl group of cysteine resides plays an important role in the hydrolysis of VLCFA-CoA, although it has not been clarified whether the cysteine residues have a catalytic role at present. The superfamily of ACOTs is divided into two groups, Type-I and Type-II. Type-I ACOTs belong to the α/β-hydrolase superfamily and utilize a catalytic triad of serine, histidine and aspartic acid. Type-II ACOTs are defined by the structural motif called the HotDog domain^36^. As HotDog-fold enzymes lack conserved catalytic residues, a variety of catalytic resides and mechanisms exist^37^. However, ABCD1 has no homology of consensus motifs in the active site in either Type-I or Type-II. A recent study of CTS in *A. thaliana* suggested that the amino acid residues Asp863, Gln864 and Thr867 in transmembrane helix 9 compose a catalytic triad based on the putative structure of CTS obtained by the remodeling of the ABCB10 structure and comparison of human hotdog-fold thioesterase 2 (hTHEM2)^30^. In the case of mouse cytosolic acyl-CoA thioesterase (CTE-I), the catalytic triad is constituted by serine, histidine and aspartic acid. Interestingly, when the active-site serine is replaced by cysteine, the ACOT activity is reduced to 1.9% of wild type CTE-I and the mutant protein is strongly labeled with ^14^C-palmitate due to a marked decrease in the hydrolysis rate. The covalently bound fatty acid is removed by treatment with neutral hydroxylamine^38^. Similar cases are reported in other thioesterses^39–41^. As the ACOT activity of ABCD1 is quite low compared with these ACOTs^42–44^, a cysteine residue instead of serine might form active site in the ACOT of ABCD1. ABCD1 possesses three cysteine residues in its TMD, Cys39, Cys88 and Cys121. The structural model of ABCD1 that specifies the position of the transmembrane and coupling helices had been proposed based on the 2.85 Å resolution crystal structure of the mitochondrial ABC transporter ABCB10^31^. In the model, Cys39 and Cys88 are located on the N-terminal cytosolic region followed by transmembrane helix 1, and Cys121 is located on peroxisome luminal loop between transmembrane helix 1 and 2. It is suggested that the fatty acid moiety of VLCFA-CoA enters to substrate-binding pocket of ABCD1, since the fatty acid moiety is hydrophobic and CoA moiety is hydrophilic. It might be difficult for Cys121 to access CoA moiety of VLCFA-CoA. Therefore, it is assumed that Cys39 or Cys88 are responsible for ACOT activity of ABCD1. We have to further investigate to identify which cysteine residue is responsible for ACOT activity. In addition, to clarify other members composing catalytic triad is also needed.

Concerning the substrate recognition of ABCD1, the 3′-phosphate group of acyl-CoA is important (Fig. 7A). It has been demonstrated that the 3′-phosphate group of acyl-CoA plays a significant role in human medium-chain acyl-CoA dehydrogenase^45,46^. In this case, the 3′-phosphate group makes an enthalpic contribution derived from van der Waals interactions between the 3′-phosphate group and the surrounding protein moiety. The crystal structure of human ABCD1 is needed for a proper understanding of the positional relationship between the active site of ABCD1 and the 3′-phosphate group of acyl-CoA substrates.

We must consider the reasons why such a transport mechanism involving the hydrolysis of VLCFA-CoA is required. The cleavage of acyl-CoA into fatty acid and CoA by ABCD1 results in a loss of high-energy bond and the consumption of additional ATP is required for the activation (re-thioester formation) of free fatty acid with CoA. VLCFA-CoA possesses very hydrophobic fatty acid and hydrophilic CoA. Since it is postulated that it is difficult to translocate large, amphipathic molecular compounds across biological membranes, the hydrolysis of VLCFA-CoA might be necessary for transport. In the transport system which we propose, another enzyme acyl-CoA synthetase is required before the β-oxidation of VLCF-CoA within the peroxisomes. In the case of *S. cerevisiae*, the knockout of Faa2p and/or Fat1p reduced VLCF β-oxidation, and this suggests the enzymes are coupled together for the function of Pxa1p and Pxa2p^19,47^. The LCFA-CoA synthetases LACS6 and LACS7 in *A. thaliana* were initially suggested to be involved in LCFA β-oxidation^22,48^. It was recently demonstrated by co immunoprecipitation with an anti-CTS antibody that these proteins are associated with CTS^23^, suggesting they are coupled with CTS during the transport of acyl-CoA into peroxisomes. In mice, the knockout of the *Vlcs* gene, encoding very long chain fatty acyl-CoA synthetase (VLCS), resulted in a significant decrease of VLCFA β-oxidation^49^. Moreover, it is reported that human ABCD1 physically interacts with VLCS^50^.

In our transport assay, only a modest amount of NBD-C16 was transported into ABCD1-liposomes when compared to the total amount of hydrolyzed NBD-C16. Other components, such as the fatty acyl-CoA synthetase(s), which are coupled with ABCD proteins on the inner surface of peroxisomal membrane or the acyl-CoA binding domain containing 5 (ACBD5)^51^ might be required during the transport of the fatty acid moiety of VLCFA-CoA into peroxisomes. In addition, the transporters for CoA and ATP must also be required for the activity of acyl-CoA synthetase(s) before VLCFA β-oxidation in peroxisomes. SLC25A17 was identified as the mammalian transporter for CoA into peroxisomes^52^. The transport of the reconstitution of several components including ABCD1 should be examined. In addition, how the transport of CoA is coupled to ABCD1, and especially whether ABCD1 and the CoA transporter form a complex remain as future challenges.

In this study, we identified the unique characteristics of the ACOT domain of the human ABCD1 protein and showed its importance during transport of VLCFA-CoA into peroxisomes. Furthermore, we directly demonstrated that ABCD1 transports the fatty acid moiety after the hydrolysis of VLCFA-CoA.

## Materials and methods

### Yeast strains and culture conditions

*Komagataella phaffii* gene-disrupted strain *pxa1*Δ *his4* was used as the host strain to express the human ABCD1 proteins. The strains were grown in YPD (1% yeast extract, 2% peptone, and 2% glucose) or BM (0.5% yeast extract, 0.5% methanol) supplemented, when required, with Zeocin (100 µg/ml) at 30 °C.

### Disruption of the *PXA1* gene in *K. phaffii*

The oligonucleotide primers used for the PCR reactions are listed in supplementary Table S1. A 1.1-kb upstream region and a 1.6-kb downstream region of *KpPXA1* were amplified by PCR with primer sets Fw-Kppxa1-5′/Rv-Kppxa1-5′ and Fw-Kppxa1-3′/Rv-Kppxa1-3′, respectively, using genomic DNA of *K. phaffii* SMD1168 as the template. Similarly, a 1.5-kb fragment encoding the Zeocin resistance gene was amplified with the primers Fw-zeo + 5 and Rv-zeo + 3 using pPICZ as the template. These three fragments were fused by the PCR fusion technique with the primers Fw-Kppxa1-5′ and Rv-Kppxa1-3′^53^. The resulting fragment was transformed into the *K. phaffii* SMD1168. Disruption of the *KpPXA1* gene was confirmed by Southern blot analysis with *Afl*III-digested genomic DNA of the transformant and a 1.6-kb fragment from the downstream region of the *KpPXA1* gene as the probe (Fig. S1).

### Construction of hABCD1 expression plasmids

The plasmid for expressing His-tagged ABCD1 was constructed as described previously^25^. Next, the plasmids for expressing ABCD1(K513A) were constructed as follows. A 7.6-kb fragment was amplified by inverse PCR with the primer sets Fw-ABCD1-K513A/Rv-ABCD1-K513A using pIB4-His-ABCD1 as the template, and then this fragment was self-ligated using T4 Polynucleotide Kinase (Toyobo, Osaka, Japan) and Ligation high (Toyobo) to form pIB4-His-ABCD1(K513A). The plasmids for expressing ABCD1(aa.1‒431) were constructed by the same procedure as described above. The mutated *ABCD1* sequence was confirmed using the BigDye Terminator v3.1 Cycle Sequencing Kit (Applied Biosystems, Foster City, CA) in an ABI Prism 3500 sequencer (Applied Biosystems).

### Purification and reconstitution of ABCD1

ABCD1 expressed in *K. phaffii* was purified as previously described^23^ with some modification. Yeast cells were grown to mid-log phase on YPD medium. Subsequently, the cells were transferred to BM medium and incubated for 12 h at 30 °C. The cells were resuspended with Tris buffer (50 mM Tris–HCl pH7.5, 300 mM NaCl, 5 mM DTT) and then disrupted with 0.3-mm zirconia beads in a Multi-Beads Shocker (YASUI KIKAI Co., Ltd, Osaka, Japan). All following purification steps were conducted at 4 °C. Undisrupted cells, nuclei and other cell debris were removed by centrifugation at 1500×*g* for 10 min. The resulting supernatant (cell-free extract) was subjected to centrifugation at 14,000×*g* for 30 min to obtain an organelle pellet. Membranes were solubilized by 0.5% β-DDM for 3 h on an end-over-end rotator. Insoluble material was removed by centrifugation at 100,000×*g* for 30 min. The supernatant was incubated with cOmplete His-Tag Purification Resin (Roche, Basel, Switzerland) with 5 mM imidazole on an end-over-end rotator for 16 h at 4 °C. Subsequently, the resin was washed twice with Tris buffer containing 0.1% β-DDM and 50 mM imidazole, and ABCD1 was eluted with Tris buffer containing 0.1% β-DDM and 500 mM imidazole. Protein concentration in eluate fraction was determined by the method of Bradford. As His-ABCD1 and the non-specific protein existed in the eluate fraction, the amount of His-ABCD1 was calculated from ration of the intensity of His-ABCD1 and the non-specific protein in acrylamide gel after SDS-PAGE and CBB staining.

Liposomes were prepared as previously described^54^. Soybean L-α-phosphatidylcholine (10 mg/mL, Type II-S; Sigma, St. Louis, MO) was suspended in buffer containing 20 mM Tris–HCl (pH 7.5). The mixture was sonicated until it became clear in a bath-type sonicator and then frozen and thawed five times. Liposomes were stored at − 80 °C until use. Aliquots of 50 μg of eluate fraction were mixed with 500 μg of liposomes and then frozen and thawed twice. The mixture was diluted 30-fold with reconstitution buffer containing 20 mM Tris–HCl pH 7.5 and 0.5 mM DTT. Reconstituted proteoliposomes were pelleted via centrifugation at 150,000×*g* for 1 h at 4 °C and then suspended in 200 μl of 20 mM Tris–HCl pH 7.5. The amount of ABCD1 incorporated into liposomes was calculated by immunoblot analysis of His-ABCD1 using His-ABCD1 in eluate fraction as standard. The signal strength of His-ABCD1 was quantified by the image analysis software Image J.

### ATPase activity

The ATPase activity of ABCD1 reconstituted into liposomes was determined by measuring the release of inorganic phosphate from ATP as described previously^55^. ABCD1-liposomes were mixed with reaction buffer (50 mM Tris–HCl pH7.6, 300 mM NaCl, 11 mM MgCl_2_, 1.1 mM EGTA, 2.2 mM DTT, 10 mM sodium azide, 2 mM ouabain) in the presence of 10 mM AlF_3_ when indicated and incubated for 5 min at 37 °C. The reaction was started by the addition of ATP (final conc. 5 mM) and incubated at 37 °C.

### Thioesterase activity

The Acyl-CoA thioesterase activity of reconstituted ABCD proteins was measured with fluorometry using NBD-palmitoyl-CoA (NBD-C16-CoA) (Avanti Polar Lipids, Alabaster, AL) as the substrate as described previously^23^. ABCD1-liposomes were incubated with 2 μM NBD-C16-CoA and 1 mM MgCl_2_ at 37 °C in a total volume of 50 µl. Ten µl aliquots were removed at various times and the reaction was immediately stopped by the addition of 30 µl cold acetone and 5 µl of this mixture was subjected to TLC to separate NBD-C16-CoA and NBD-palmitic acid (NBD-C16). At the analytical stage, the mobile phase composition 1-butanol/water/acetic acid (7:2:1) was selected and optimized using pre-coated glass-backed silica gel plates (Merck, Darmstadt, Germany). The NBD fluorescence was detected by ImageQuant LAS4000 mini biomolecular imager (GE Healthcare, Buckinghamshire, England) using blue fluorescence light (excitation: 460 nm; emission: Y515Di filter). The amount of hydrolyzed NBD-C16 was quantified by the image analysis software Image J using the standard curve of NBD-C16 (Fig. S10) and the activity was calculated.

### Uptake of NBD-C16 into ABCD1-liposomes

The reaction mixture contained ABCD1-liposomes in 100 µl of buffer (20 mM Tris–HCl pH 7.5, 10 μg/ml BSA (fatty acid free), 2 µM NBD-C16-CoA, 1 mM MgCl_2_, 1 mM ATP). After incubation at 37 °C, 100 µl of 10 mM sodium dithionite solution in 20 mM Tris–HCl pH10 was added to quench the remaining NBD-C16-CoA. Then the ABCD1-liposomes were precipitated by centrifugation 15,000 × *g* for 30 min at 4 °C and the supernatants were removed. The pellet was resuspended by 20 µl of 80% acetone and sonicated, and 5 µl of this solution was subjected to TLC. NBD-C16-CoA and NBD-C16 were detected as described above after the separation by TLC. The flow chart of transport assay is shown in Fig. S9A. To hydrolyze the nonspecifically embedded NBD-C16-CoA into liposomes, the pellet was resuspended with 20 mM Tris–HCl pH 7.5 and incubated with 1 N NaOH for 30 min on ice in a total volume of 30 µl. Then the mixture was neutralized with 4 µl of 6 N HCl. The reaction mixture was subsequently subjected to TLC with acetone.

### Acylation and deacylation of ABCD1

ABCD1-liposomes that had been incubated with 5 μM NBD-C16-CoA and 1 mM MgCl_2_ at 37 °C were subjected to SDS-PAGE and the gel was fixed using methanol. To test the stability of the interaction between NBD-labelled compound with ABCD1, the gel was further incubated with 1 M hydroxylamine pH 7.0, 0.1 N KOH in 20% methanol, 0.1 N HCl, or as a control, 1 M Tris–HCl pH 7.0 for 18 h at room temperature. The NBD fluorescence was detected as described above.

### Preparation of NBD-labeled compounds

NBD-C16-3′-dephosphoCoA was prepared as follows. NBD-C16-CoA was incubated with Nuclease P1 (FUJIFILM Wako, Osaka, Japan) in 50 mM Tris–HCl pH 8.0 and 1 mM DTT at 37 °C for 3 h, then subjected to TLC. After fluorometry, the corresponding position of NBD-C16-3′-dephosphoCoA on the silica gel plate were scraped, and the compound was eluted from silica gel using water. The eluate was applied to a Sep-Pak C_18_ cartridge column (Waters, Milford, MA). The column was eluted with 70% acetonitrile after washing with 20% acetonitrile. Purified NBD-C16-3′-dephosphoCoA was evaporated using a Savant SpeedVac concentrator and dissolved in water.

NBD-hexanoyl-CoA (NBD-C6-CoA) was prepared as follows. NBD-hexanoic acid (NBD-C6) was incubated with His-*Kp*FAA2 from *K. phaffii* in 20 mM Tris–HCl pH7.5, 5 mM CoA, 5 mM ATP, 5 mM MgCl_2_ and 1 mM DTT at 30 °C for 3 h, then subjected to TLC analysis. The synthesized NBD-C6-CoA was purified as described above.

## Supplementary Information

Supplementary Information.
