# Supplementary material for: Acyl-CoA thioesterase activity of peroxisomal ABC protein ABCD1 is required for the transport of very long-chain acyl-CoA into peroxisomes
Source: Sci Rep. 2021 Jan 26;11:2192. doi: 10.1038/s41598-021-81949-3 (PMC7838297; doi:10.1038/s41598-021-81949-3)

Article

Title:

Acyl-CoA thioesterase activity of peroxisomal ABC protein ABCD1 is required for the transport of very long-chain acyl-CoA into peroxisomes

Author names and affiliations:

Kosuke Kawaguchi[1*], Emi Mukai[1], Shiro Watanabe[2], Atsushi Yamashita[3], Masashi Morita[1], Takanori So[1] and Tsuneo Imanaka[4]

[1]Graduate School of Medicine and Pharmaceutical Sciences, University of Toyama, 2630 Sugitani, Toyama 930-0194, Japan.

[2] Institute of Natural Medicine, University of Toyama, 2630 Sugitani, Toyama 930-0194, Japan.

[3]Faculty of Pharma-Sciences, Teikyo University, 2-11-1 Kaga, Itabashi-Ku, Tokyo 173-8605, Japan

[4]Faculty of Pharmaceutical Sciences, Hiroshima International University, 5-1-1 Hirokoshinkai, Kure, Hiroshima 737-0112, Japan

[*]Correspondence and requests for materials should be addressed to K.K.

E-mail: kkawa@pha.u-toyama.ac.jp

Phone: +81-76-434-7547

Supplementary Materials and Methods

Orientation of ABCD1 reconstituted into liposomes

ABCD1-liposomes were incubated with or without trypsin, or with trypsin in the presence of 0.1% Triton X-100 for 60 min on ice. Samples were immediately subjected to SDS-PAGE and followed by immunoblot analysis using anti-ABCD1 (clone 1AL-2B4, Merck, Darmstadt, Germany) recognizing aa.495–648. The intensity of the bands corresponding ABCD1 and its fragments was quantified by the image analysis software Image J.

Turnover of ABCD1 labeled by NBD-C16-CoA

ABCD1-liposomes were incubated with NBD-C16-CoA for 30 min at 37°C as transport assay. To remove excess NBD-C16-CoA, the reaction mixture was applied to EconoSpin Gel Filtration Column (Ajinomoto Bio-Pharma, Osaka, Japan) equilibrated with 20 mM Tris-HCl pH 7.5 and the eluate containing ABCD1-liposomes was recovered by centrifugation at 800 x *g* for 2 min at 4°C. Then, ABCD1-liposomes labeled with NBD-C16 were incubated with 5 μM palmitoyl-CoA in 20 mM Tris-HCl pH 7.5, 10 μg/ml BSA (fatty acid free), 1 mM $MgCl_2$, and 1 mM ATP at 37°C. Aliquots were removed at 10 min and 20 min, and subjected to SDS-PAGE. The NBD fluorescence was detected as described in the section of acylation and deacylation of ABCD1 in Materials and Methods.

Construction of KpFAA2 Expression Plasmids

The oligonucleotide primers used for PCR reactions are listed in supplementary Table S1. The plasmid for expressing His-tagged KpFAA2 was constructed as follow. The 5.7-kb fragment containing *AOX1* promoter and His-tag was amplified by inverse PCR with primer sets Fw-inv-pIB4-His/Rv-inv-pIB4 using pIB4-His-ABCD1 as the template. The 2.2-kb fragment coding *KpFAA2* was amplified with the primer set Fw-infu-FAA2/Rv-infu-FAA2 using genomic DNA of *K. phaffii* SMD1168 as the template. These two fragments were fused using an In-Fusion PCR

cloning kit (TaKaRa, Shiga, Japan) to yield pIB4-His-KpFAA2.

Purification His-KpFAA2

Purification of His-KpFAA2 was performed as described previously [1] with some modification. Organelle pellet was obtained by the same procedure for His-ABCD1. All following purification steps were conducted at 4°C. Membranes were solubilized by 1% Triton X-100 for 3 h on an end-over-end rotator. Insoluble material was removed by centrifugation at 100,000 x *g* for 30 min. The supernatant was incubated with cOmplete His-Tag Purification Resin (Roche, Basel, Switzerland) on an end-over-end rotator for 16 h at 4 °C. Subsequently, the resin was washed 2 times with Tris buffer containing 50 mM imidazole, and His-FAA2 were eluted with Tris buffer containing 500 mM imidazole.

Reference

1 Okamoto, T. *et al.* Characterization of human ATP-binding cassette protein subfamily D reconstituted into proteoliposomes. *Biochem biophys res com* **496**, 1122-1127 (2018).

Supplementary Table 1 List of oligonucleotide primers

Primer name	5'- sequence-3'
Fw-Kppxa1-5'	GGAATTCCCACAGCGAAAGATTCACCG
Rv-Kppxa1-5'	GATACCGCTCGCCTAAAGACGTCAAAGAGG
Fw-Kppxa1-3'	CTGGAGACCAGAAGAAAATACTTATAGATTAGCACTC
Rv-Kppxa1-3'	CTCTCCAGGAAGAAGGATCGCAGGC
Fw-Zeo+5'	GACGTCTTTAGGCGAGCGGTATCAGCTCACTCAAAGG
Rv-Zeo+3'	GTATTTTCTTCTGGTCTCCAGCTTGCAAATTAAAGCCTTCGAGCG
Fw-ABCD1-K513A	CCCCAATGGCTGCGGCGCGAGCTC
Rv-ABCD1-K513A	CCCGTGATGAGCAGATGCATGCCTTCC
Fw-ABCD1-1-431	CTCGAGCTGCAGGCATGCAAGCTTC
Rv-ABCD1-1-431	GTTAGCGCTGAACATCTTCAAATACCTGG
Fw-infu-FAA2	CCGAATTGCGTCTAGAATGTCACATCTCAAAAAGATCCAG
Rv-infu-FAA2	GATGATGATGGTCGACCTACATCTTGGTCTCCCTGAGAAG
Fw-inv-pIB4	GACCAAGATGTAGGTCGACCATCATCATCATCATC
Rv-inv-pIB4-His	GAGATGTGACATTCTAGACGCAATTCGGTGGTGG

Supplementary Figure Legends

Supplementary Figure 1 Disruption of the *PXA1* gene in *K. phaffii*. Construction of the *pxa1*Δ strain was performed and the disruption of the gene was confirmed. (A) Physical map of the cloned fragment and disruption strategy. (B) Southern analysis was conducted for *Afl*III-digested DNA extracted from the *PXA1*-deleted *K. phaffii* strain together with the wild type strain. A 1.6-kb downstream region of *KpPXA1* was amplified by PCR with the primer set Fw-Kppxa1-3/Rv-Kppxa1-3 using genomic DNA of *K. phaffii* SMD1168 as the template was used as the hybridization probe.

Supplementary Figure 2 Orientation of ABCD1 reconstituted into liposomes. (A) Proteoliposomes containing ABCD1 (1.02 μg) were incubated with or without 10 ng of trypsin (lines 1 and 2) or with trypsin in the presence of 0.1% triton X-100 (line 3) for 60 min on ice. ABCD1 was detected by immunoblot analysis using anti-ABCD1 recognizing aa.495–648. (B) The amount of ABCD1 and its fragments was quantified using the image analysis software Image J. Signal intensity of ABCD1 without trypsin treatment has been normalized to 1. Error bars indicate the standard error (n=3).

Supplementary Figure 3 Preparation of the negative control. *K. phaffii* SMD1168 *pxa1*Δ, the host strain of heterologous ABCD1 expression, was used to prepare the purified fraction by the same procedure as the purification of His-ABCD1, and a non-specific protein in the eluate fraction was reconstituted into liposomes. Each fraction was subjected to SDS-PAGE and CBB staining. The asterisk indicates a non-specific protein.

Supplementary Figure 4 Effect of inhibitors on ABCD1 ACOT activity. (A) ABCD1-liposomes were incubated with NBD-C16-CoA and 1 mM of each compound for 30 min at 37°C. The aliquots were subjected to TLC. (B) ACOT activity was measured in the presence of various concentrations of *p*CMB for 30 min at 37°C. The aliquots were subjected to TLC.

Supplementary Figure 5 Esterase from porcine liver hydrolyzes NBD-C16-3’-

dephosphoCoA. NBD-C16-CoA and NBD-C16-3'-dephosphoCoA (NBD-C16-dePiCoA) were incubated with or without esterase from porcine liver for 30 min at 37°C. The aliquots were subjected to TLC.

Supplementary Figure 6 Purification of the *Kp*FAA2 expressed in *K. phaffii*. His-tagged *Kp*FAA2 was expressed in *K. phaffii* under the control of the *AOX1* promoter and purified using the same procedure as the purification of His-ABCD1 except for the solubilization step. Triton X-100 was used to solubilize His-*Kp*FAA2 instead of β-DDM. Protein samples were resolved via SDS-PAGE followed by CBB staining (upper panel) and immunoblot (lower panel) using an anti-His antibody. The asterisk indicates a non-specific protein.

Supplementary Figure 7 Effect of ATP on ABCD1 ACOT activity. ABCD1-liposomes were incubated with NBD-C16-CoA in the presence of various concentrations of ATP for 30 min at 37°C. The aliquots were subjected to TLC.

Supplementary Figure 8 Purification and reconstitution of ABCD1(a.a.1-431) and ABCD1(K513A) (A) Purified and reconstituted His-ABCD1(a.a.1-431) and His-ABCD1(K513A) were subjected to SDS-PAGE, and the gels were stained with CBB. The asterisk indicates a non-specific protein. (B) ATPase activities of reconstituted ABCD1s were measured. Proteoliposomes containing ABCD1 wild type, K513A or negative control liposomes containing non-specific protein were incubated with 5 mM ATP for 30 min at 37°C and the phosphate that was released was measured. Error bars indicate the standard error (n=3).

Supplementary Figure 9 Non-specific detection of NBD-C16-CoA transport into liposomes. (A) The scheme of transport assay. (B) Liposomes without protein were incubated with NBD-C16-CoA at 37°C for the indicated periods. After incubation, the remaining NBD-C16-CoA was quenched with sodium dithionite. Subsequently, ABCD1-liposomes were precipitated by centrifugation and then resuspended with 80% acetone. NBD-C16 and NBD-C16-CoA were separated by TLC. The fact that NBD-C16

is not detected indicates that NBD-C16 is absent from the region inside the liposomes. The detection of NBD-C16-CoA was not dependent on the incubation, suggesting non-specific binding to the liposomes. (C) Turnover of ABCD1 labeled by NBD-C16-CoA. ABCD1-liposomes were incubated with NBD-C16-CoA for 30 min at 37°C and then collected using gel filtration column. Subsequently, ABCD1 labeled with NBD-C16 on liposomes were incubated with non-labeled palmitoyl-CoA. Aliquots were removed at various times and subjected to SDS-PAGE and the acylation of ABCD1 with NBD-C16-CoA was analyzed (upper panel). His-ABCD1 contained in each fraction was detected by immunoblot (lower panel). (D) Liposomes containing ABCD1 or the non-specific protein were incubated with NBD-C16-CoA at 37°C. After incubation, the remaining NBD-C16-CoA was quenched with sodium dithionite. Subsequently, the liposomes were precipitated by centrifugation and resuspended with 20 mM Tris-HCl pH 7.5. Then the liposomes were incubated with 1 N NaOH for 30 min on ice to hydrolyze the NBD-C16-CoA embedded on the outer surface of the liposomes. After incubation, mixtures were neutralized with HCl and subjected to TLC. The detection of NBD-C16-CoA completely disappeared, indicating the hydrolysis of NBD-C16-CoA. These results suggest that the thioester linkage of NBD-C16-CoA is exposed to the outer surface of the liposomes and the NBD-C16 moiety embeds in the outer leaflet of the liposomes.

Supplementary Figure 10 Standard curve to quantify NBD-labeled compounds. Five μl of samples containing various amount of NBD-C16 were subjected to TLC. The NBD fluorescence was detected by ImageQuant LAS4000 mini biomolecular imager and quantified by the image analysis software image J. The exposure time was set to 5 sec for detecting ACOT activity (A) or 30 sec for detecting transport activity (B).

Supplementary Figure 11 Full length images of each figure.

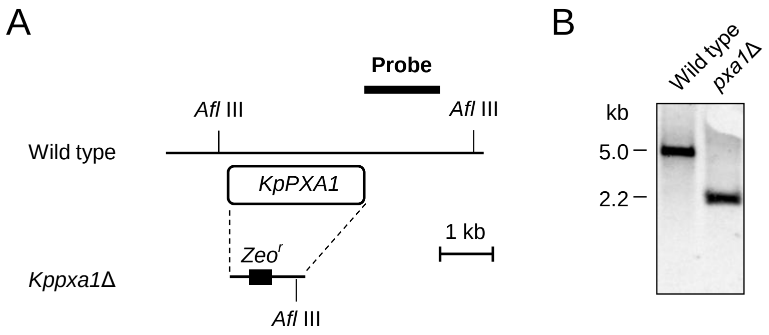

Kawaguchi et al. Figure S1

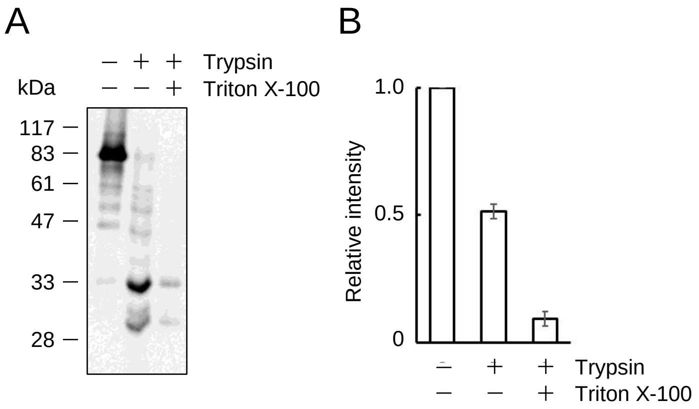

Kawaguchi et al. Figure S2

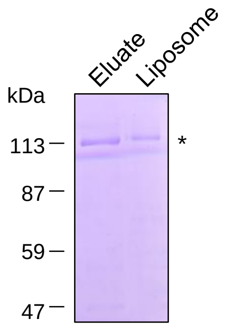

Kawaguchi et al. Figure S3

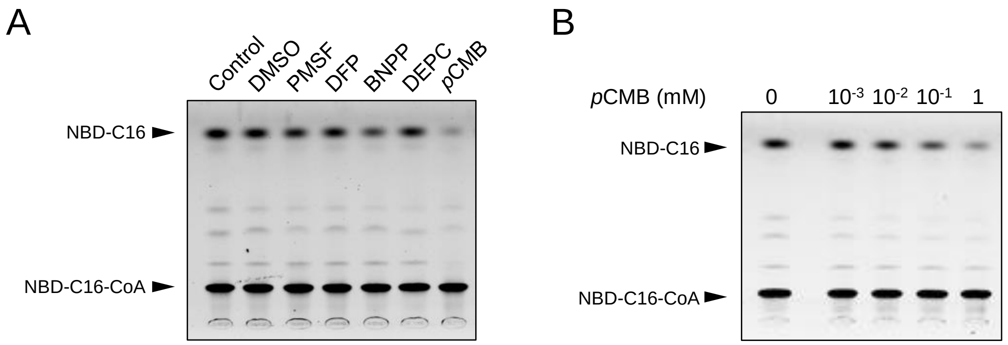

Kawaguchi et al. Figure S4

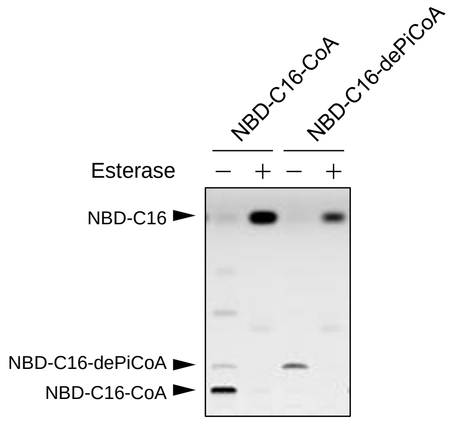

Kawaguchi et al. Figure S5

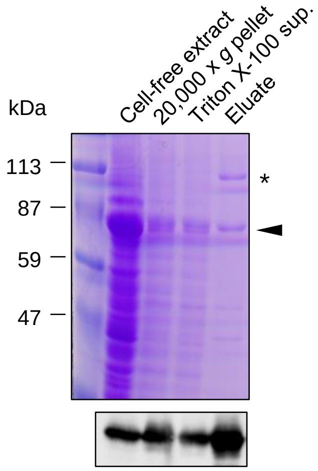

Kawaguchi et al. Figure S6

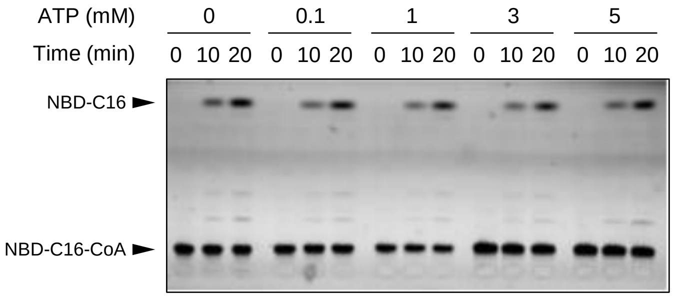

Kawaguchi et al. Figure S7

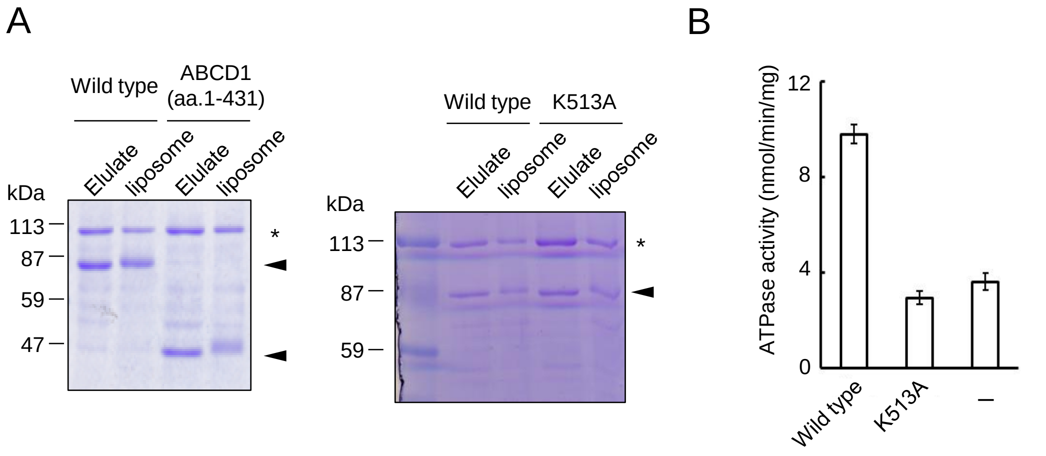

Kawaguchi et al. Figure S8

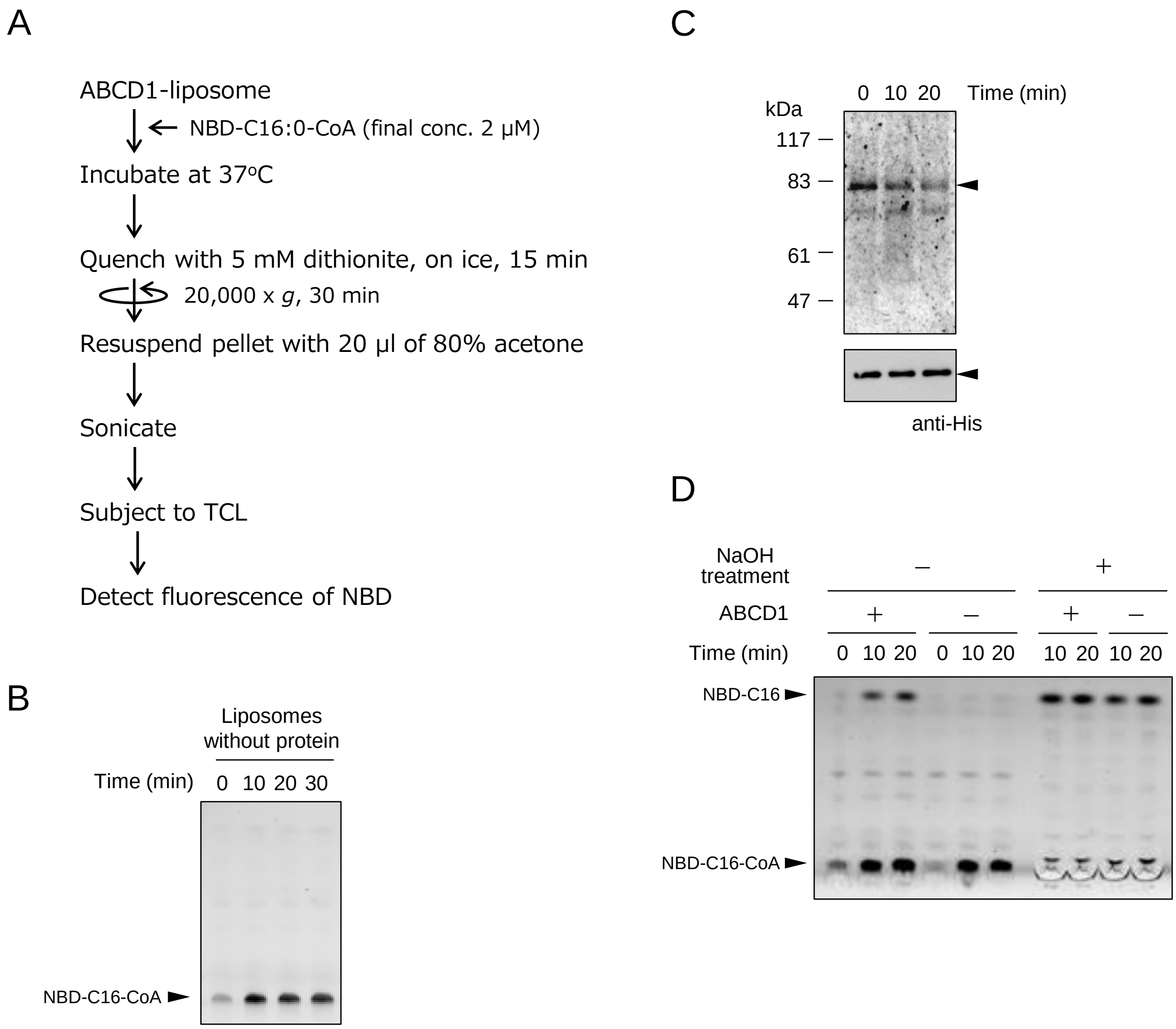

Kawaguchi et al. Figure S9

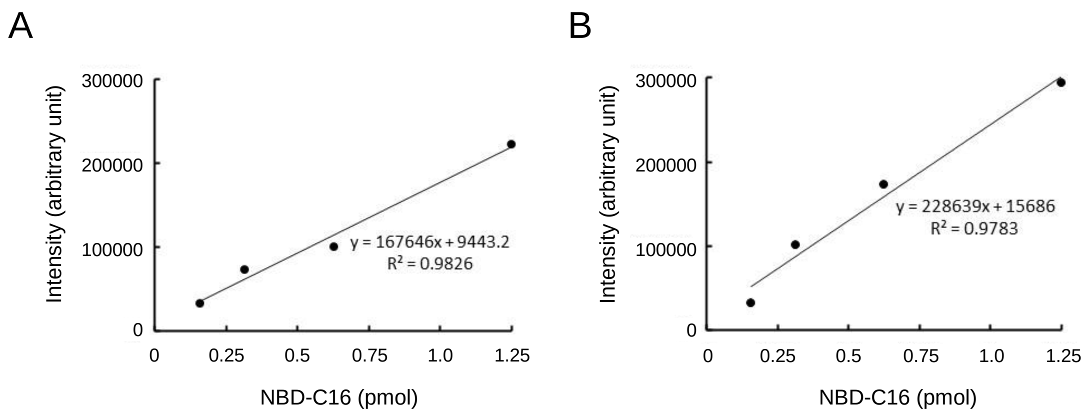

Kawaguchi et al. Figure S10

Figure 1A

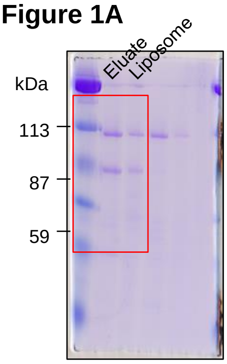

Figure 2A

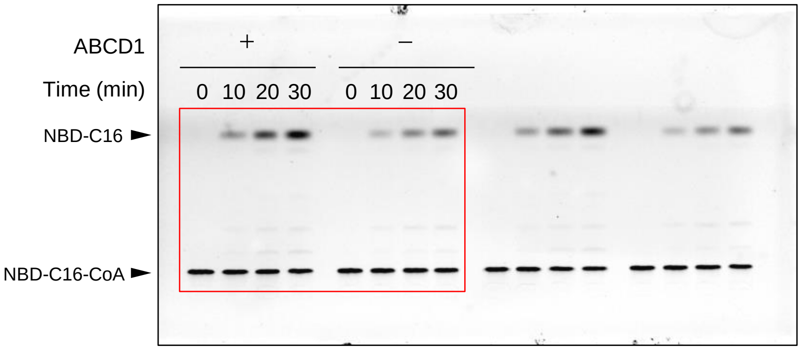

Figure 2C left panel

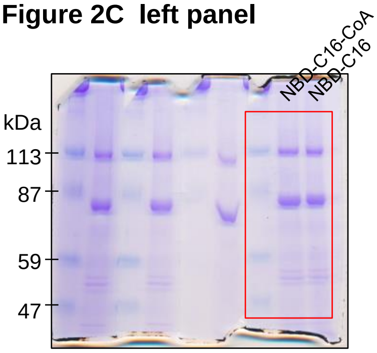

Figure 2C right panel

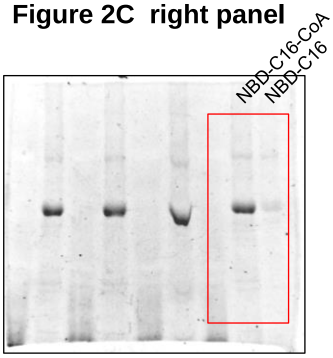

Figure 3A

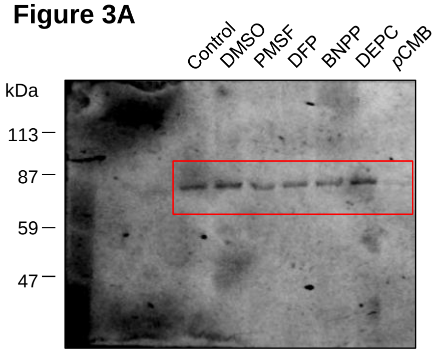

Figure 3C

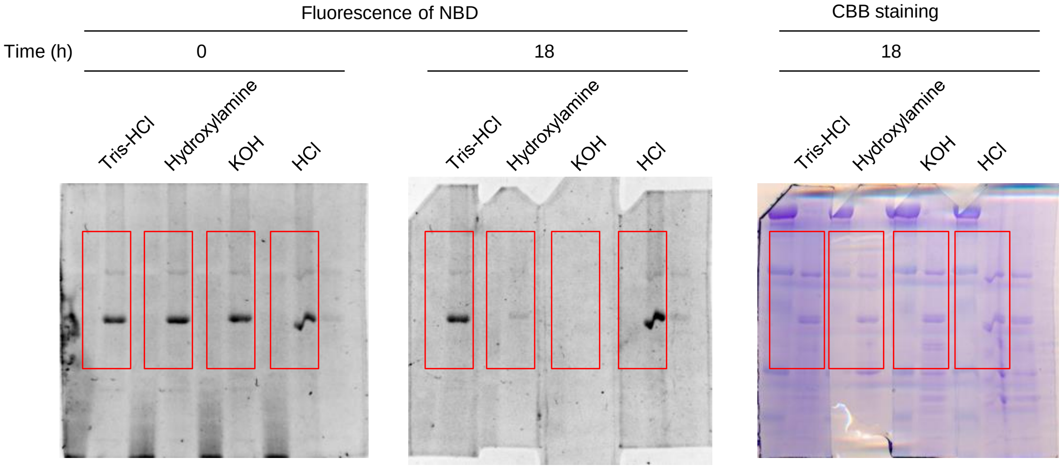

Figure 4A and S5

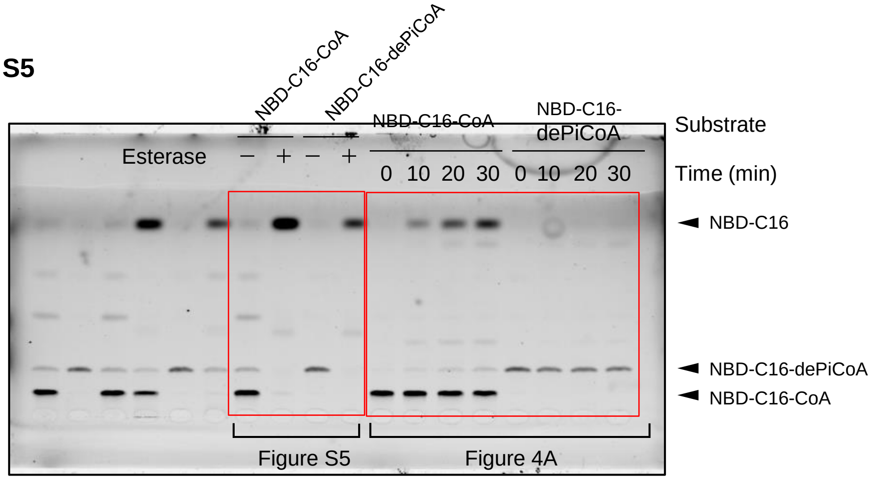

Kawaguchi et al. Figure S11

Figure 4B

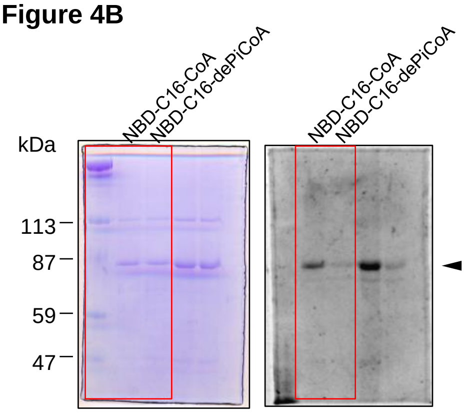

Figure 4C

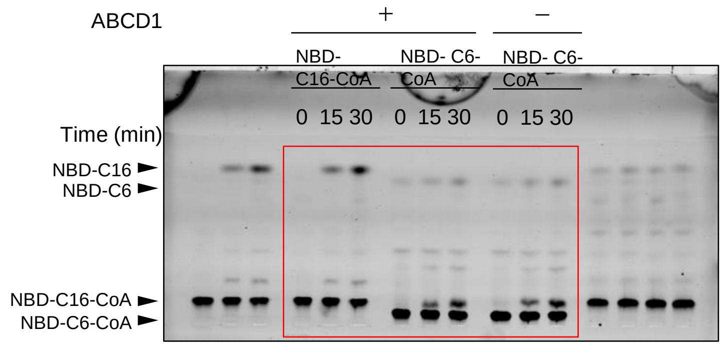

Figure 4D

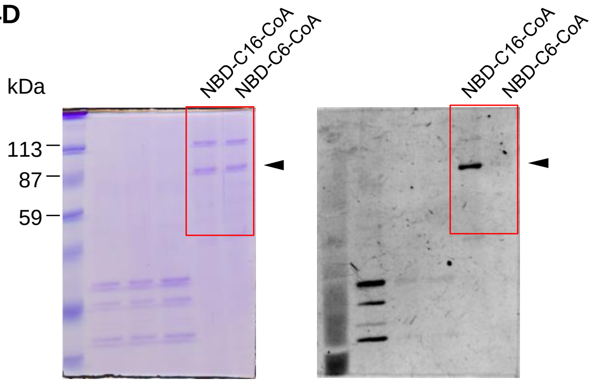

Figure 5B

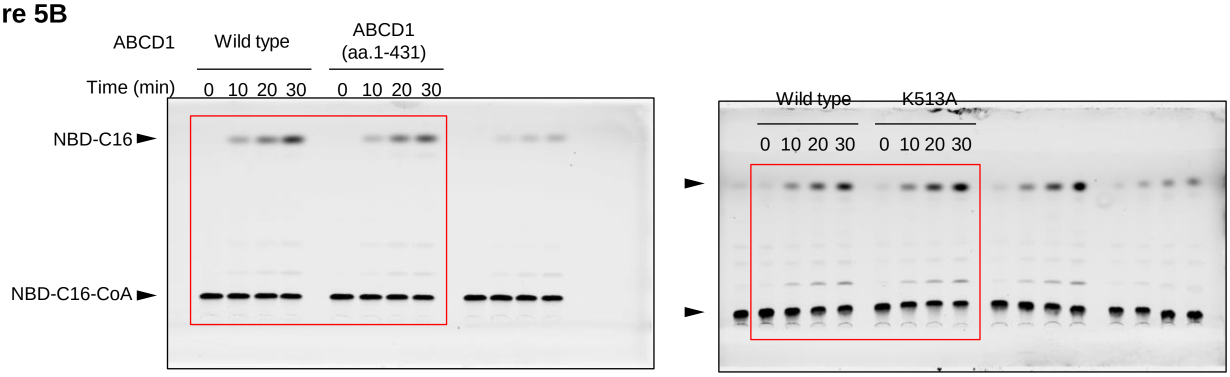

Figure 5D

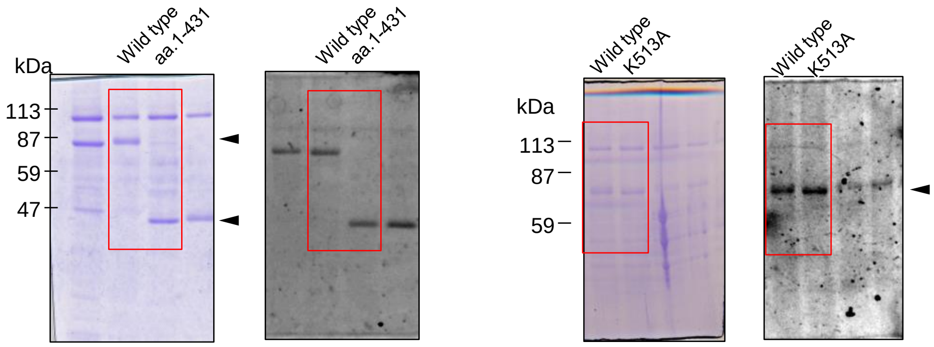

Figure 6A

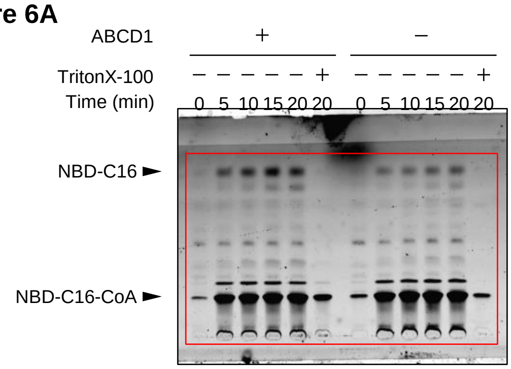

Figure 6C

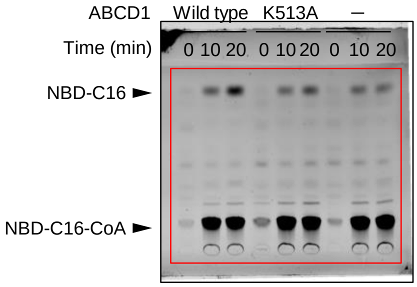

Figure 6B

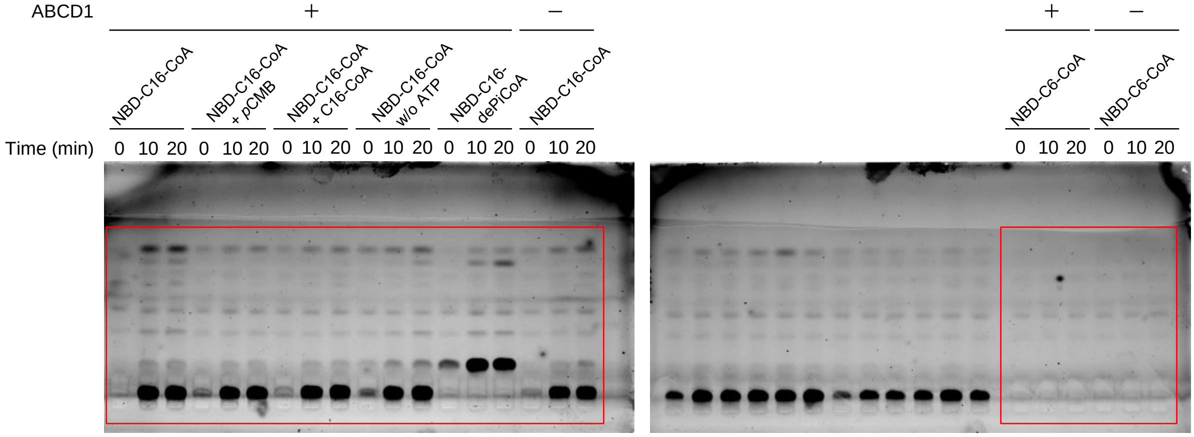

Figure S1

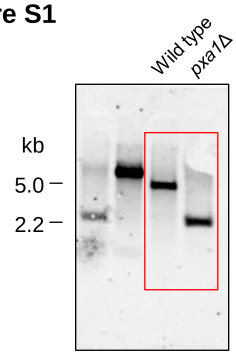

Figure S2A

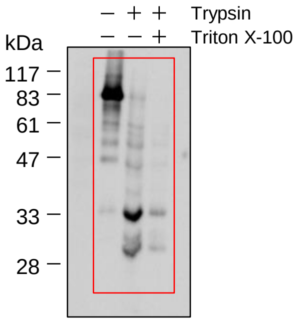

Figure S3

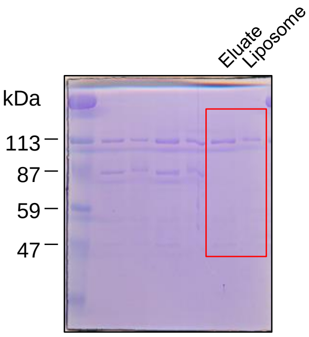

Figure S4A

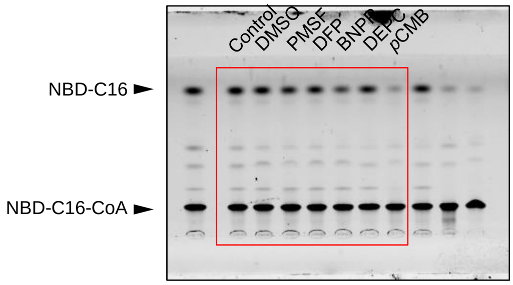

Figure S4B

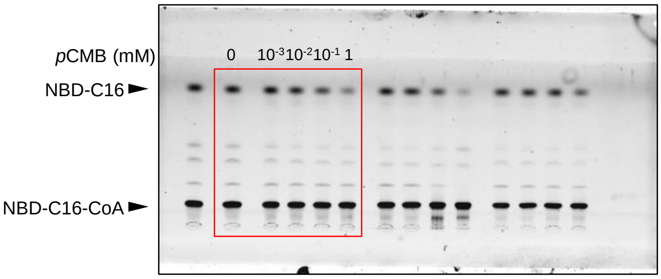

Figure S6

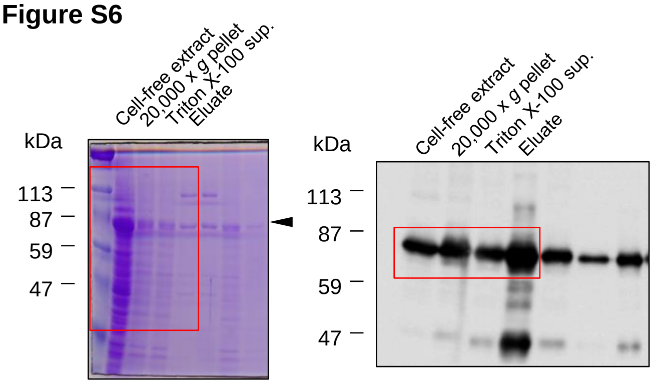

Figure S7

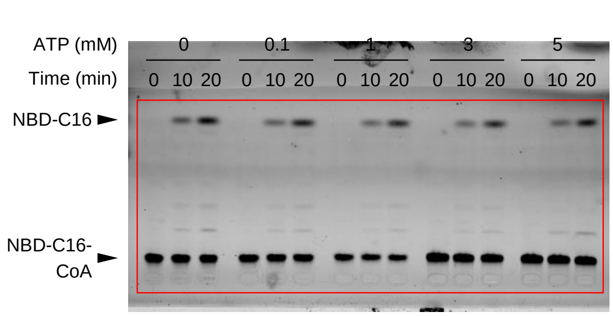

Figure S8A

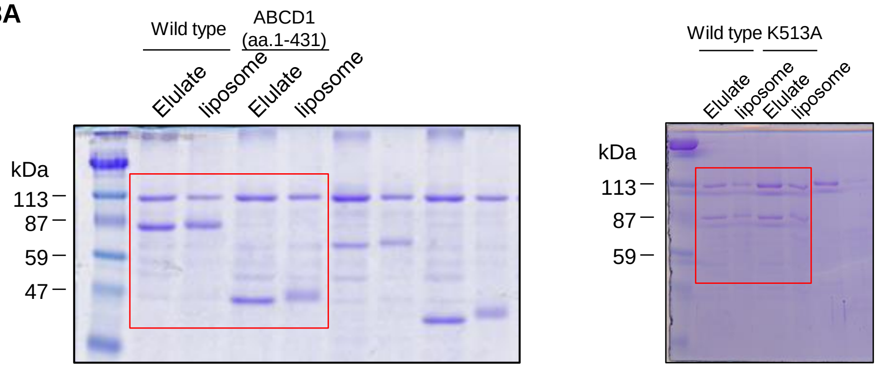

Figure S8B

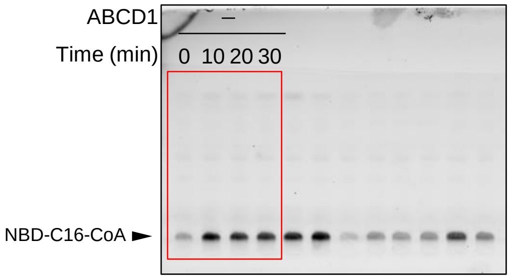

Figure S8C upper panal

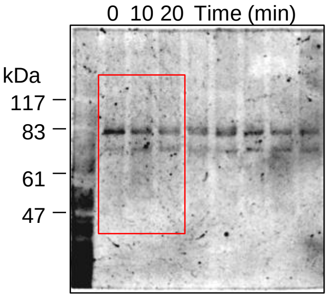

Figure S8C lower panal

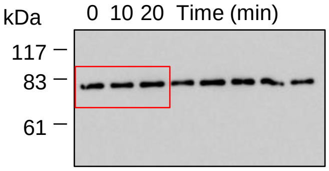

Figure S8D

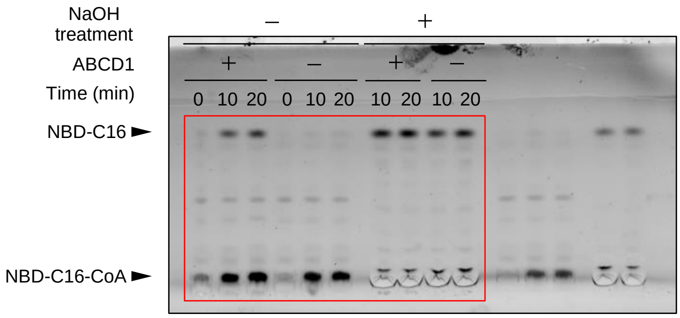

Supplement: Supplementary file 1 — Supplementary Information. [file 41598_2021_81949_MOESM1_ESM.pdf]
